# A bio-inspired adaptive model for search and selection in the Internet of Things environment

**DOI:** 10.7717/peerj-cs.762

**Published:** 2021-12-01

**Authors:** Soukaina Bouarourou, Abdelhak Boulaalam, El Habib Nfaoui

**Affiliations:** 1 Computer Science Department, Faculty of Sciences Dhar EL Mahraz, Sidi Mohamed Ben Abdellah University, Fez, Morocco; 2 Computer Science Department, National School of Applied Sciences, Sidi Mohamed Ben Abdellah University, Fez, Morocco

**Keywords:** IoT, Sensor, Context properties, WhaleCLUST, TOPSIS, Clustering, Service search, Sensor selection

## Abstract

The Internet of Things (IoT) is a paradigm that can connect an enormous number of intelligent objects, share large amounts of data, and produce new services. However, it is a challenge to select the proper sensors for a given request due to the number of devices in use, the available resources, the restrictions on resource utilization, the nature of IoT networks, and the number of similar services. Previous studies have suggested how to best address this challenge, but suffer from low accuracy and high execution times. We propose a new distributed model to efficiently deal with heterogeneous sensors and select accurate ones in a dynamic IoT environment. The model’s server uses and manages multiple gateways to respond to the request requirements. First, sensors were grouped into three semantic categories and several semantic sensor network types in order to define the space of interest. Second, each type’s sensors were clustered using the Whale-based Sensor Clustering (WhaleCLUST) algorithm according to the context properties. Finally, the Technique for Order of Preference by Similarity to Ideal Solution (TOPSIS) was improved to search and select the most adequate sensor matching users’ requirements. Experimental results from real data sets demonstrate that our proposal outperforms state-of-the-art approaches in terms of accuracy (96%), execution time, quality of clustering, and scalability of clustering.

## Introduction

**Figure 1:**
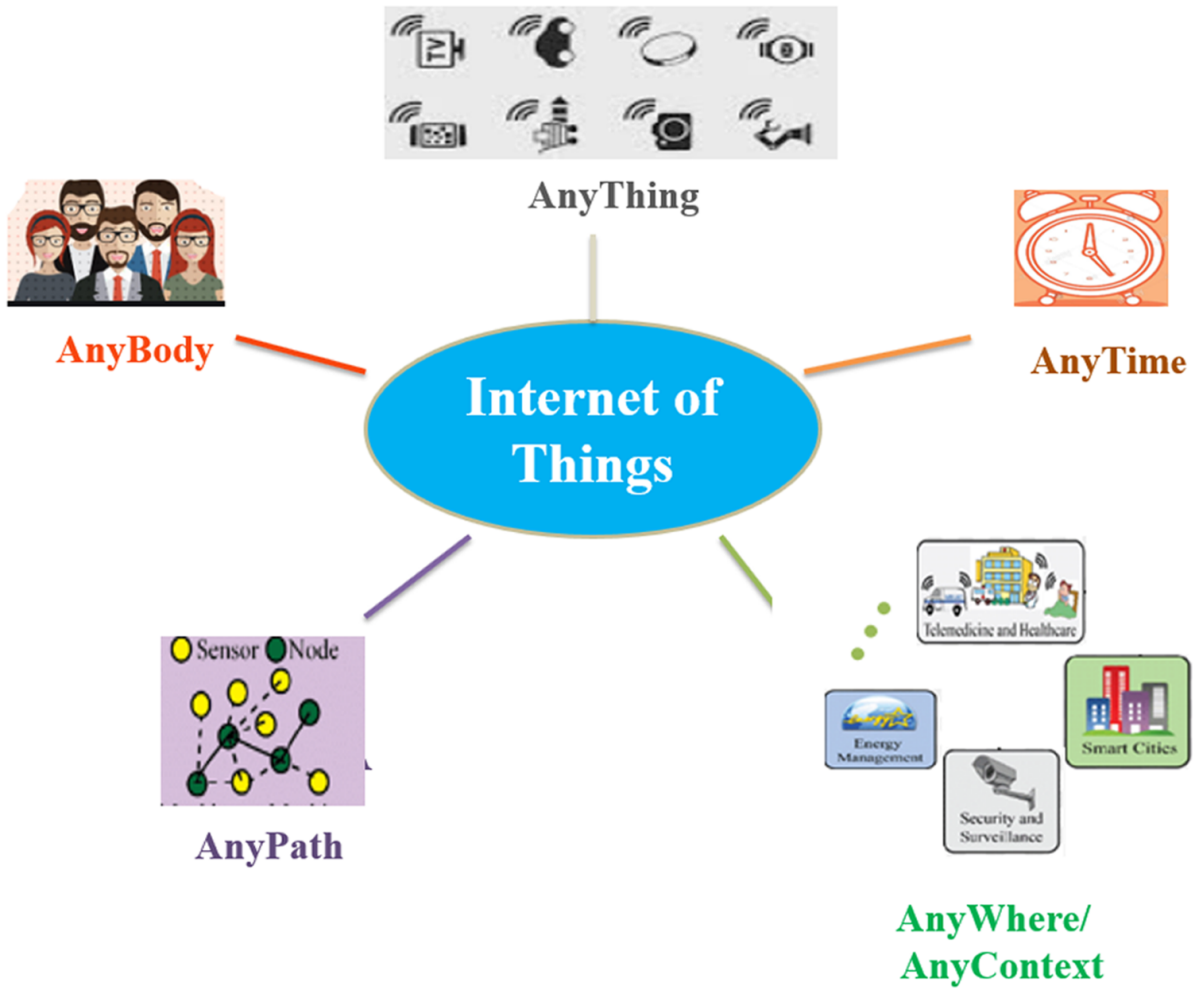
The definition of IoT.

The Internet of Things (IoT) is a technology used to connect people and physical devices (sensors, actuators, Radio-Frequency Identification (RFID), *etc*.) *via* the Internet, while continuously collecting and sharing data (Fig. 1) (Perera et al., 2013b). This interconnection defines three types of interactions: people to people, people to things/machine, and things/machine to machine/things (Guillemin & Friess, 2009).

**Figure 2:**
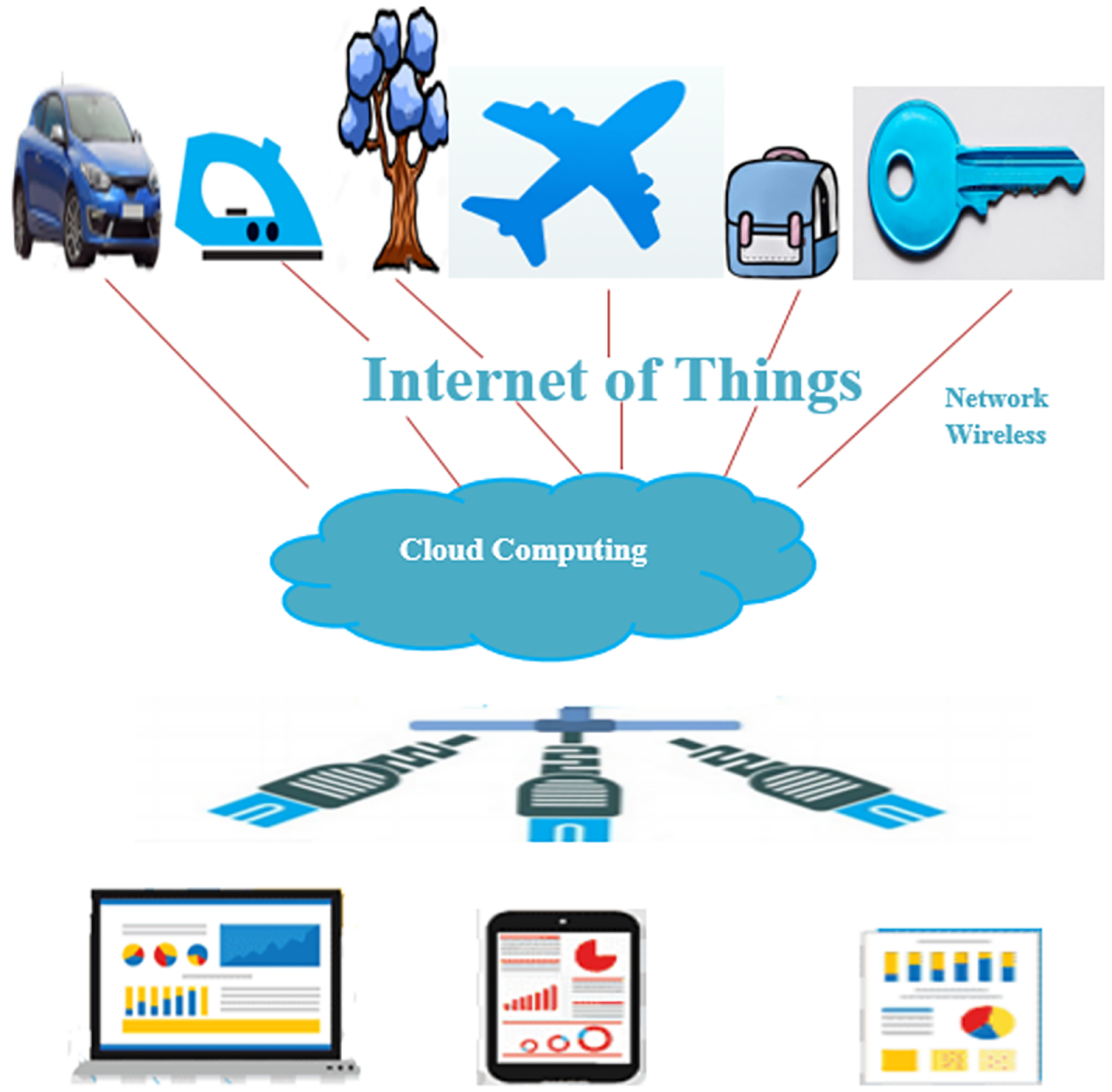
The integration of IoT and web services.

The integration of the IoT and the web (Web of Things) had led to the creation of new kinds of services and applications in different domains: remote healthcare (Kadhim et al., 2020), indoor air quality (Saini & Dutta, 2020), vehicular traffic management (Lalitha & Pounambal, 2020), and air pollution monitoring (Arora et al., 2019). Figure 2 illustrates this integration. These applications have sought to create a smart environment between the real and the virtual that makes energy, transport, cities, and other areas more intelligent in order to enhance human life (Patel & Patel, 2016).

A market analysis company group predicted that there will be over 100 billion devices connected the IoT by 2025 (Global Sensors in Internet of Things (IoT) Devices Market, 2016–2022, https://www.bisresearch.com/industry-report/global-sensors-in-iot-devices-market-report-forecast.html). There will be a significant impact worldwide as the number of connected IoT devices that communicate, sense, and share information grows. These interconnected devices generate a large amount of data, which must then be collected, analyzed, and used; this demonstrates we have an enormously searching space (Zhang, Yang & Huang, 2011).

Different interconnected sensors may be especially useful for monitoring and recording environmental phenomena such as temperature, sound, pollution levels, humidity, and wind (Abdmeziem, Tandjaoui & Romdhani, 2016). Therefore, it is important to determine which sensors/devices should be selected to retrieve the desired data. This wireless sensor network suffers from various limitations, including unbalanced energy consumption, hardware malfunction, deliberate attacks, physical localization, and weak security (Laouid et al., 2017, 2019; Mahmood, Seah & Welch, 2015; Saoudi et al., 2017). It is impossible to collect all data from all of the existing sensors in the network due to the increasing number of networks. Thus, our main goal is to determine which sensors should be selected, taking into consideration of required processing time and overall accuracy.

We sought to overcome these limitations with an efficient approach to searching for and selecting the appropriate sensors for a query with minimal time consumption and a high quality. The proposed architecture consists of categorizing all current sensors in the network into three semantic categories (SCs); Society, Industry, and Environment. In parallel, for each semantic category, several semantic sensor network types (SSNTs) were created, and then the Whale-based Sensor Clustering Algorithm was applied to cluster the sensors using their contextual information. The Technique for Order of Preference by Similarity to Ideal Solution (TOPSIS) was then used to select the appropriate sensors for the user query.

The remainder of this article is organized as follows: “Related works” is devoted to a description and review of the most studies focused on sensor search and selection techniques. The “Preliminaries” section includes a definition of the problem, the WhaleCLUST Algorithm, and the TOPSIS Algorithm. “Proposal” presents the proposed model on suitable way formulation. “Experiments” discusses the main implementation results. Finally, “Conclusion” provides a concluding summary and future work.

## Related works

Existing methods used to address sensor search issues can be categorized into content-based methods that look for sensors that generate such data, and context-based methods that use the sensors properties.

### Content-based approaches

Content-based sensor searches can be conducted by processing and clustering the data generated by the different sensors, respectively. The aim is to obtain the optimal subset of devices that provide the desired data at any instance of time.

Bu (2018) proposed a fuzzy c-means approach to process and analyze data handling in IoT by which the data are grouped into clusters using the canonical decomposition method, which reduces the attributes of each object before loading the dataset into the memory. This approach enhances clustering efficiency and optimizes execution time, although it employs only a limited number of attributes to evaluate data (Tang & Fong, 2018).

A meta-heuristic algorithm for clustering and automatic clustering on big IoT data was proposed by Tang & Fong (2018). This partitioned clustering method optimizes the mini-batch and parallel partition-based Dynamic Group Clustering (DGC) for the IoT big data environment. The method is comprised of three activities: first, a sample of the dataset is partitioned into mini-batches; then the centroids of the mini-batches of data are adjusted; lastly, the mini-batches are collated to form clusters. This method maximizes the quality of the clusters related to the choice of the optimal batch size.

Another meta-heuristic algorithm for clustering and automatic clustering on big IoT data was suggested by Jabeur et al. (2020). This approach has highlighted the impact of spatial events on IoT clustering. The performed clustering for IoT depends on the following six main features: location, energy, connectivity, users’ requirements, communication, and semantics based on the Firefly Algorithm (FICA). This approach is inconsistent in its efficient selection of CH; however, it has registered more energy consumption and a high complexity.

Elahi et al. (2009) introduced a predictive model by calculating the estimation probability. Their model can find sensors satisfying a user query by predicting the current resources’ real data. However, this model is limited in that it can be used only on sensors that present periodic patterns and, therefore, is not highly accurate.

Ostermaier et al. (2010) developed a real-time search engine for the Web of Things named DYSER. This predictive engine assists in finding real-world entities using statistical models to make predictions based on their real-time state and assists in the discovery of resources with a limited number of sensor data retrievals.

Truong, Römer & Chen (2012) used the fuzzy set to calculate a similarity score between two candidate sensors, which were then used to obtain a ranked list of matching sensors. This method becomes time-consuming when the number of sensors within the network is increased.

Tables 1 and 2 summarize and compare different content-based and context-based search techniques with the following parameters: number of used sensors, processing time, attributes or properties, accuracy, and dynamicity (*e.g*., removal or connection of new sensors in the sensor network).

**Table 1:** Functional and non-functional comparison of content-based search approaches.

Researches	Number of sensors	Properties	Execution time	Accuracy	Dynamicity
Elahi et al. (2009)	250	Unlimited	–	–	Unconsidered
Ostermaier et al. (2010)	385	Unlimited	–	–	Unconsidered
Truong, Römer & Chen (2012)	42	Unlimited	–	–	Unconsidered
Bu (2018)	Unlimited	Limited	650 min/64 Clusters	0.94/64 Clusters	Considered
Tang & Fong (2018)	12,000	100	1.20E+00	–	Considered
Jabeur et al. (2020)	800	Unlimited	–	–	Unconsidered

**Table 2:** Functional and non-functional comparison of context-based search approaches.

Researches	Number of sensors	Number of properties	Execution Time(s)	Accuracy	Dynamicity
Aberer, Hauswirth & Salehi (2007)	100,000	2	–	–	Unconsidered
Nath, Liu & Zhao (2007)	100,000	2	–	–	Unconsidered
Le-Phuoc et al. (2011)	100,000	2	–	–	Unconsidered
Perera et al. (2013a)	1,000,000	6	9.5	98%	Unconsidered
Hsu, Lin & Chen (2014)	50	1	600	–	Unconsidered
Ebrahimi et al. (2017)	1,000,000	6	8	92%	Considered
Nunes et al. (2017)	10,000	6	–	–	Unconsidered
Nunes et al. (2018)	209,555	6	785	–	Unconsidered
Kertiou et al. (2018)	1,000,000	6	9	95%	Unconsidered
Babu, Prathap & Samuel (2019)	20	4	2,009	91.99%	Unconsidered
Singh, Baranwal & Tripathi (2020)	100	9	2.85	–	Unconsidered
Zannou, Boulaalam & Nfaoui (2021)	3,000	4	–	–	Unconsidered

### Context-aware approaches

The content-based method summarizes the processing of all collected data obtained from an enormous number of connected sensors in an IoT network; it is not practical to treat all the collected data, leading many researchers to choose context-based method approach. Context-awareness is an efficient technique for selecting data that requires additional processing and attention.

Context is the characterization of an object in an environment at any place and time; this is extracted from the expression of one or more IoT resources (Abowd et al., 1999). Below, we present an overview of works focused on a sensor search based on context properties.

The Global Sensor Networks (GSN) (Aberer, Hauswirth & Salehi, 2007) is a data-processing engine aimed at providing flexible middleware to address sensor data integration and distributed query processing. This approach allows registering sensors with their meta-data in an XML structure. The query is a text-based search; however, this engine features ambiguous descriptions of sensors that users add. Similar to GSN model, the Microsoft SensorMap (Nath, Liu & Zhao, 2007) enables the user to choose appropriate sensors based on their location, type, and keywords.

Ebrahimi et al. (2017), Kertiou et al. (2018), Le-Phuoc et al. (2011), Perera et al. (2013a), (2013b) addressed the issue of using the Semantic Sensor Network (SSN) Ontology (Compton et al., 2012) as the basis for conceptual modeling in the IoT domain (Janowicz et al., 2010). Among these works, Linked Sensor Middleware (LSM) (Le-Phuoc et al., 2011) is a platform that pairs real-world data with the Semantic Web in a unified model, where raw data are saved in relational databases, and the database schema is mapped to the ontology for improvement. This platform, however, offers limited functionalities for searching based on logical queries. For example, querying the resources relies on selecting services based only on the sensor location and its type, assuming that the obtained data are static and not subject to frequent changes.

A context-aware sensor search selection and ranking model called CASSARAM (Perera et al., 2014; Perera et al., 2013a, 2013c) suggests collecting point-based and proximity-based demands from users before plotting them in a multidimensional space. This method employs a distributed search by executing parallel processing over different server nodes to collect local high-ranking sensors. This collection and management of context-properties is a challenging approach for a large number of sensors due to its disregard for system performance in dealing with changes (add or delete) in the sensor network.

Ebrahimi et al. (2017) was inspired by the behavior of ants when developing the Ant-Clustering (AntCLUST) algorithm to cluster sensors based on their contextual information in the form of Sensor Semantic Overlay Networks (SSONs). The search queries are transmitted to each SSON to select the clusters that contain the most appropriate sensors. Although this strategy is meant to maintain its performance against dynamicity in the IoT, the system suffers from vulnerability to dynamicity issues and a time-consuming off-line computing phase.

Nunes et al. (2017) proposed a resource discovery and selection process based on the required parameters. The authors evaluated and compared the overall quality of this selection among three multi-objective decision methods: the Simple Additive Weight method (SAW), TOPSIS, and VIseKriterijumska Optimizacija I Kompromisno Resenje (VIKOR) with the Pareto optimal solutions.

The Elimination-Selection (ES) model (Nunes et al., 2018) combined the Fast-Non-Dominated sort algorithm and the multiple-criteria decision method TOPSIS to enhance quality and response time. The selections given by TOPSIS were reordered and chosen as the prominent best option by using a Fast-Non-Dominated sort, which was applied in an agricultural case study (Nunes et al., 2016) using a real dataset. The ES model increased the proportion of the non-dominated selections and increased the processing time by tens of times.

Hsu, Lin & Chen (2014) proposed an architecture allowing sensor search and selection based on identification of the sensors’ characteristics, such as sensing range, accuracy, or residual energy among a large set of available sensors. SSN ontology was used to represent the properties of the sensor and this architecture has three components: server, gateway, and sensor. The user posts a request to the server that is connected to different gateways to offer a response. In the simulation study, however, they focused only on the network lifetime.

Kertiou et al. (2018) based their proposed architecture on work by Hsu, Lin & Chen (2014), which employed the context information of sensors with dynamic skylines operators to decrease the search space and select the best sensors depending on the user request. This requires use of distributed gateways connected to a server, where each gateway responds to the users’ local requests. There is lower time complexity of the dynamic skyline algorithm, but it does require users to input the ideal values of sensor properties (Zheng et al., 2019).

A comparison of the above-mentioned search techniques (context-based and content-based approaches) is summarized in Table 3, including the search approach, system architecture, data, algorithm/ontology, and prototype/simulation.

**Table 3:** Comparison of different search techniques approaches.

Approach	Search publication	Search approach	Architecture	Data used	Algorithm/ontology used	Prototype/simulation
Content-based sensor	Elahi et al. (2009)	Time-related search	Centralized	ETH & MERL	Single and multi-period prediction model	Simulation
	Ostermaier et al. (2010)	Real-time search	Centralized	Bicing	Aggregated prediction model	Web-based prototype
	Truong, Römer & Chen (2012)	Event-based search	Centralized	NOAA, IntelLab , MavHome	Fuzzy logic	Web-based prototype
	Bu (2018)	Time-related search	Distributed	eGSAD, sWSN (Zhang, Chen & Leng, 2015)	Fuzzy c-means algorithm	Simulation
	Jabeur et al. (2020)	Spatiotemporal-based search	Distributed	Randomly generated	Bio-inspired algorithms	Java-based prototype
Context- aware sensor	Perera et al. (2013a)	Context-based search	Distributed	Phenonet project, LSM project, and Bureau of Meteorology	SSN Ontology and Apache Jena SDB & TDB	Java-based prototype
	Ebrahimi et al. (2017)	Context-based search	Centralized	LSM project, Bureau of Meteorology, MesoWes and AirQuality sensor dataset	SSN Ontology (Compton et al., 2012)	Simulation
	Nunes et al. (2017, 2018)	Context-based search	Centralized	Open Weather Map	–	Simulation
	Hsu, Lin & Chen (2014)	Event-based search	Centralized	ViSIoT	SSN Ontology (Compton et al., 2012)	Web-based prototype
	Kertiou et al. (2018)	Context-based search	Centralized	LSM Project, Bureau of Meteorology, and Phenonet project	SSN Ontology (Compton et al., 2012)	Simulation

Babu, Prathap & Samuel (2019) presented a different context-aware reliable sensor selection method in the IoT environment. The proposed method senses secure data from a sensor that fulfills the user requirements. It also utilizes methods including user addition, context specification, location sensing, user context counting, reliable information storage in the cloud, and the ability to select the provider's best results. However, this method requires a more accurate sensor selection model based on context information and user priority for cyber-physical systems (CPS) such as smart farming, and smart cities.

Singh, Baranwal & Tripathi (2020) proposed an IoT framework for conducting the selection process in QoS-Aware Selection of IoT services, which assumed the sensor, the network, and application layers. The multi-criteria decision making (MCDM) was applied as a combination of two methods under the analytic hierarchy process (AHP) and Technique for Order Preference by Similarity to Ideal Solution (TOPSIS). The AHP method was used to measure the weight of the QoS criteria, and TOPSIS ordered the services. These methods define the QoS parameter based on three potential IoT components: things, computing entity, and communication entity. Without regard to illustrating the adaptability framework, they presented only the selection part of the service composition.

With the same aims as content-based and context-aware methods, Zannou, Boulaalam & Nfaoui (2021) paired sensor nodes. In this, the best pairing creates a better search for a required service in an optimal path and takes into regard the residual energy. This approach was only applied when the search process for a specific service was demanded by a sensor node in a social network.

There are several architectures and methods that may be used to search for a service based on the content and the context of sensors. However, each study has its restrictions, as mentioned above. The mechanisms to resolve a search task are specific to a given goal. Here, we propose an architecture consisting of several gateways distributed in the network and managed by a server. We applied new methods on this architecture to consider their limitations while keeping an acceptable response time and a low network overload. Furthermore, the deletion or addition of sensors is a crucial challenge that should be considered in the context of a dynamic IoT environment.

### Preliminaries

This section includes the necessary background for understanding the context of our study, including the problem definition, WhaleCLUST, and TOPSIS algorithms.

### Problem definition

An IoT network is composed of a set of devices (sensors, actuators, *etc*.), gateways, and a server. These devices have a specific communication/transmission, sensing /reception ranges, and different constraints in terms of energy consumption and processing capacities. The gateways should be able to collect and process the data generated from sensors based on their constraints. Each gateway manages its sub-network and the server manages those gateways in the same manner. There are many sensors in each sub-network (gateway) that can be abstracted as services and the combination of these devices within these services is the basis of the IoT application within IoT middleware solutions.

**Figure 3:**
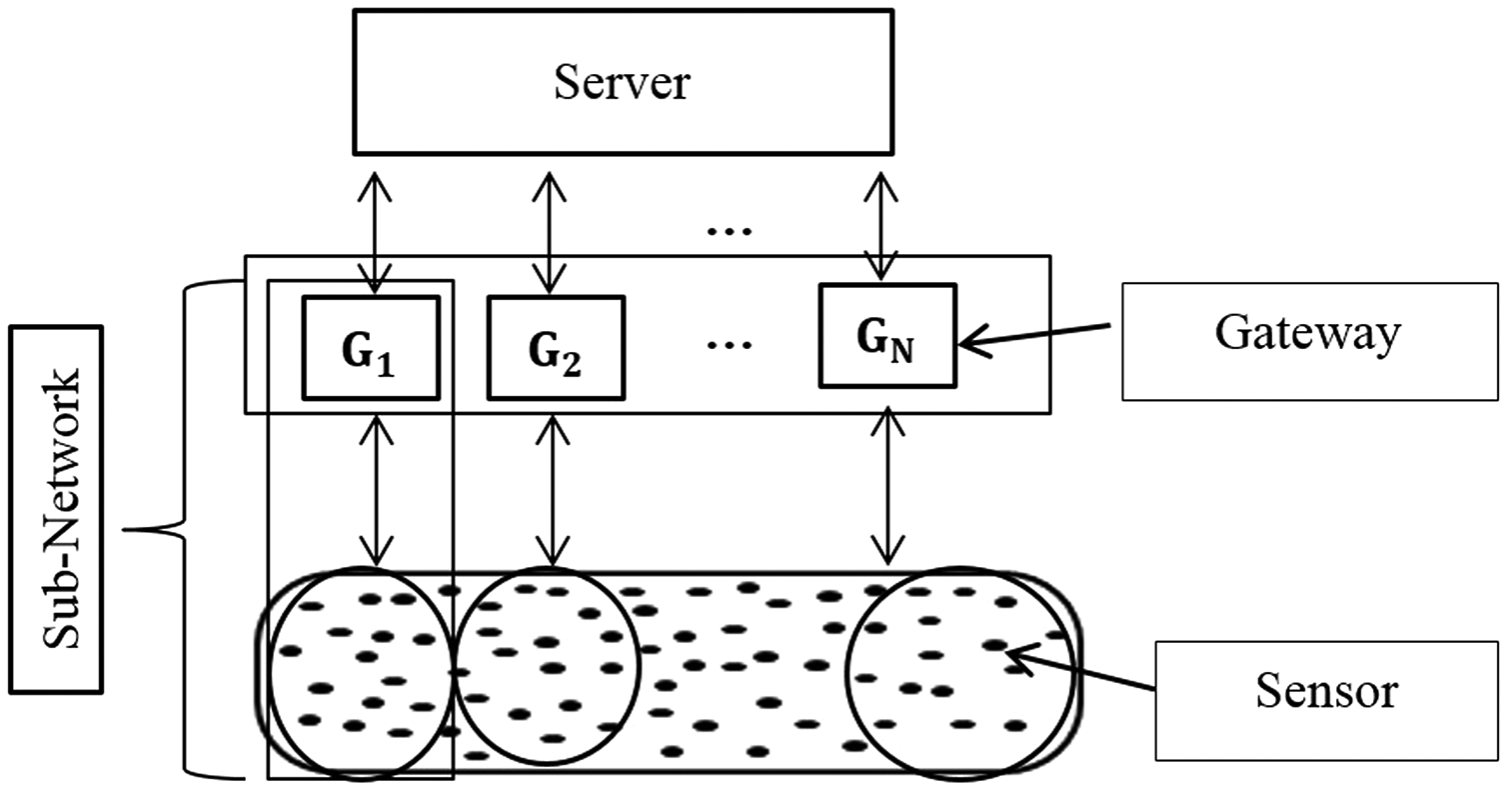
The distributed environment.

The search task for a requested service in a network is the most important functionality in IoT (Barnaghi et al., 2012) due to the huge number of sensors/services, similar services in each sub-network, and the consequently enormous amount of collected data. Thus, sensors may be clustered physically or virtually with minimal and sufficient context information. Figure 3 illustrates this distributed architecture.

When a greater number of available services are higher, the consumption will be greater; therefore, the search space must be minimized without affecting the solution quality.

Table 4 summarizes the main notations used.

**Table 4:** Notations used in the rest of this paper.

Symbol	Description
N_category_	The Number of Semantic Category (= 3).
SC_i_	The Semantic Category (i = {environment, society, industry}).
SSNT_i,j_	The Sensor Semantic Type Network of the SC_i_ and the type j.
P_h,k_	The List of index sensor of the *h*th cluster and *k*th property.
P_h,k_ (SSNT_i,j_)	The existing cluster sensors in SSNT_i,j_.
Q_j_	The Matrix of center cluster of each cluster belonging to SSNT_i,j_ for the SC_i_.
C_h,k_	Center cluster of the *h*th cluster and *k*th property.
N	The number of sensors existing in the *h*th cluster.
_Cj_	List of centers of all clusters in SSNT_i,j_.
B_center_	The best center cluster with required sensor.
BC_sensors_	All sensors existing in BC.
B_sensor_	The most similar sensor to required sensor.

### WhaleCLUST algorithm

The Whale-based Clustering Algorithm was derived from a desire to improve the Whale Optimization Algorithm (WOA) (Mirjalili & Lewis, 2016) with the principle of clustering. We defined a population as a set of search agents, and each search agent determines k centers, where k is the number of clusters. Each search agent is defined as follows:

(1) $${{\rm S}_{\rm p}} = \left( {{{\rm Z}_{{\rm p},1}},{{\rm Z}_{{\rm p},2}}, \ldots ,{{\rm Z}_{{\rm p},{\rm k}}}} \right)$$

where ${{\rm Z}_{{\rm p},\ {\rm j}}}\;$ represents the cluster center of the ${{\rm p}^{{\rm th}}}$ search agent in the cluster j.

We can represent the vector properties of a cluster center j named ${{\rm Z}_{\rm j}}$ as follows:

(2) $${{\rm Z}_{\rm j}} = \left( {{{\rm z}_{{\rm j},1}}, \ldots ,{\rm \; }{{\rm z}_{{\rm j},{\rm m}}}} \right)$$

where ${{\rm z}_{{\rm j},\ {\rm i}}}$ denotes the value of the *i*th property of the *j*th cluster center, and m is the number of properties.

**Figure 4:**
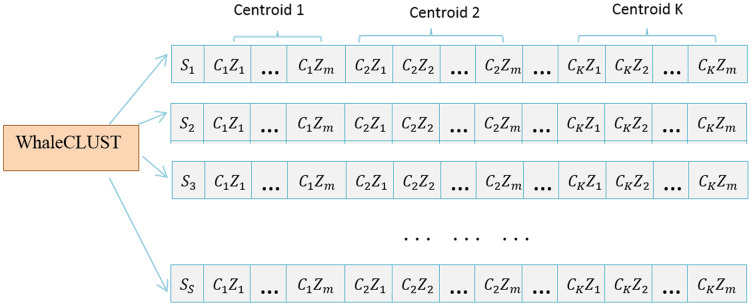
Population of S search agents at specific iterations.

Hence, a swarm (search agent) refers to several candidate centers for a given area. Figure 4 shows how a population that contains S search agents at a certain iteration t is formed.

For each search agent, object ${{\rm X}_{\rm i}}$ is first assigned to the nearest cluster center j that is verified by Eq. (3)

(3) $${\rm d}\left( {{{\rm X}_{\rm i}},{{\rm Z}_{\rm p}}} \right) = {\rm mi}{{\rm n}_{{\rm j} = 1,2, \ldots ,{\rm k}}}{\rm d}\left( {{{\rm X}_{\rm i}},{{\rm Z}_{{\rm pj}}}} \right)$$

${\rm d}\left( {{{\rm X}_{\rm i}},{{\rm Z}_{\rm p}}} \right)\;$ corresponds to the squared Euclidean distance between the vector properties of object X_i_, and the vector properties of cluster center ${\rm \; }{{\rm Z}_{\rm p}}$, as shown below in Eq. (4):

(4) $$\; {\rm d}\left( {{{\rm X}_{\rm i}},{{\rm Z}_{{\rm pj}}}} \right) = \left| {{{\rm X}_{\rm i}} - {{\rm Z}_{{\rm pj}}}} \right|$$

We computed the fitness function of each search agent ${\rm \; }{{\rm S}_{\rm p}}$, based on the distance between each cluster center j (j belongs to ${\rm \; }{{\rm S}_{\rm p}}$) and the vector properties of object ${{\rm X}_{\rm i}}$ (i belong to cluster j), by minimizing the following Euclidean distance:

(5) $${\rm fitness}\left( {{{\rm S}_{\rm p}}} \right) = \mathop \sum \limits_{{\rm j} = 1}^{\rm k} \mathop \sum \limits_{{\rm i} = 1}^{\rm N} {{\rm W}_{{\rm ij}}}{\rm d}({{\rm X}_{\rm i}},{{\rm Z}_{{\rm pj}}})$$

where k is the number of clusters/centers in search agent ${\rm \; }{{\rm S}_{\rm p}}$, and W_ij_ is the association weight of object X_i_ and cluster j, and is defined by:

(6) $${{\rm W}_{{\rm ij}}} = \left\{\matrix{1  if\ d ({\rm X}_{\rm i},{\rm Z}_{\rm pj} ) = {\rm mi}{\rm n}_{1 \le {\rm j} \le {\rm k}} d({\rm X}_{\rm i},{\rm Z}_{\rm pj} ) \\ 0  else} \right.$$

Simulating the spiral bubble-net feeding behavior process, the search agents update their position and orient themselves towards the best search agent. The description and the mathematical model for the spiral bubble-net feeding behavior process is broken down into three main aspects: encircling prey, bubble-net attacking method, and search for prey.

### Encircling prey

Humpback whales can locate the position of prey and encircle it. The algorithm considers that the existing best search agent position is the target as a prey or close to the optimum point. The other search agents will enhance their position near the best search agent. This behavior is expressed by the following equations:

(7) $$\vec S\left( {{\rm t} + 1} \right) = \overrightarrow {{S^*}} \left( t \right) - \vec A.\vec D$$

(8) $$\vec A = 2.\vec a.\vec r - \vec a$$

(9) $$\vec D = \vec C.\overrightarrow {{S^{\rm *}}} \left( t \right) - \vec S\left( t \right)$$

(10) $$\vec C = 2.\vec r$$

$\vec S\left( {{\rm t} + 1} \right)\;$ is defined as the new position of the search agent; $\overrightarrow {{S^*}} \;$ is the best search agent; ${\rm \vec A}\;$ is the coefficient vector; ${\rm \vec a}\;$ is linearly reduced within the range of 2 to 0 over the course of iterations; $\vec r\;$ is a random vector that varies in range [0, 1]; ${\rm \vec D}\;$ is the distance between the vector position of the best search agent $\overrightarrow {{S^*}} \;$ and the current position of a search agent ${\rm \vec S}\;$ at the current iteration t; ‘| |’’ is the absolute value; ${\rm \vec C}\;$ is the coefficient vector; and . is an element-by-element multiplication.

### Bubble-net attacking method (exploitation phase)

The bubble-net behavior of the humpback whale is characterized by two main mechanisms: shrinking encircling and spiral updating position. The two mechanisms have the same probabilities *p* (0.5 for each), where *p* is a random variable generated in the range [0, 1]. In the shrinking encircling mechanism, the value of $\vec a$ is decreased from two to zero over the total number of iterations in Eq. (8). The value of |A| is also decreased between -a and a. The spiral updating position mechanism is applied between prey (the best search agent) and the position of whale (the current search agent) to simulate the helix-shaped movement of humpback whale. Setting random values for |A| in the range [−1, 1], the search agent’s new position can be defined as anywhere between the original position of the current search agent and the position of the best search agent as shown below:

(11) $$\overrightarrow {{D}^{\prime}} = \overrightarrow {{S^*}} \left( t \right) - \vec S\left( t \right)$$

where $\overrightarrow {{\rm {D}^{\prime}}} \;$ is the new distance between the current search agent and the best search agent.

The new position of the current search agent was updated as follows:

(12) $$\vec S\left( {{\rm t} + 1} \right) = \overrightarrow {{D}^{\prime}} .{e^{bl}}.cos\left( {2\pi l} \right) + \overrightarrow {{S^*}} \left( t \right)$$

where b is a constant that defines the logarithmic shape, l is a random number in [−1, 1], and ‘.’ is an element-by-element multiplication.

The value of *p* is adopted to make the decision on the Equation used for updating the position of the current search agent; it is given as follows:

(13) $$\vec S\left( {{\rm t} + 1} \right) = \left\{ {\matrix{ {\overrightarrow {{S^*}} \left( t \right) - \vec A.\vec D\; \; \; \; \; \; \; \; \; \; \; \; \; \; \; \; \; \; \; \; p < 0.5} \cr {\overrightarrow {{D}^{\prime}} .{e^{bl}}.cos\left( {2\pi l} \right) + \overrightarrow {{S^*}} \left( t \right)\; \; \; \; \; \; \; \; \; p \ge 0.5\; \; \; \; \; \; \; \; \; \; \; \; \; \; } \cr } } \right.$$

### Search for prey (exploration phase)

Most meta-heuristic algorithms search for the optimum solution using a random selection. In the bubble-net method, the humpback whales randomly search for the best search agent when the values of |A| are greater than 1 or less than −1. With this consideration, the search agent moves far away from a reference whale (the best search agent chosen at first); it is expressed as follows:

(14) $$\vec S\left( {{\rm t} + 1} \right) = {\vec S_{rand}}\; - \vec A.\vec D$$

(15) $$\vec D = \vec C.{\vec S_{rand}} - \vec S$$

where ${\vec S_{rand}}$ is a random position vector.

The WhaleCLUST algorithm (Algorithm 1) initiates the search agent from a set of random solutions. The search agents update their position according to the above details.

**Algorithm 1:** The Whale-based clustering algorithm.

**Input:** S_p_ (i = 1, 2, …, s): The population set of search agent.
**Output:** S′: The best search agent
1. **Procedure** Cluster _Formulation (Si):
2. Initialize data X_i_ (i = 1, 2, …, N)
3. Choose S search agent S_p_ (i = 1, 2, …, s) to contain k randomly cluster centers
4. **for** each search agent Si
5. **for** each data vector X_m_
6. Calculate the distance using Eq. (4)
7. Assign sensors to the nearest cluster using Eq. (3)
8. **End for**
9. **End for**
10. S′: The best search agent
11. **While** t < Maximum_iteration
12.** for** each search agent
13.** **Update $\; {\rm \vec a}$**, ** ${\rm \vec A}$ , ${\rm \vec C}$**, **l and p
14.** if** p<0.5 **then:**
15.** if** |A| < 1 **then:**
16. Update search agents position using Eq. (7)
17. **end if**
18. **Else if** |A| ≥ 1 **then**:
19. Select random search agent
20. Update the position of current search agent by Eq. (14)
21. **End if**
22. **Else if** p < 0.5 **then:**
23. Use Eq.(12)
24. **End if**
25. **End for**
26. Update $\; {\rm \vec a}$, ${\rm \vec A}$ , ${\rm \vec C}$, l and p
27. Update $\overrightarrow {{\rm {S}^{\prime}}}$ if there is a better solution
28. **End while**
29. **return** S′
30. **End procedure**

### The TOPSIS algorithm

The TOPSIS algorithm is a classical MCDA method and is of great interest to researchers in the subject. The TOPSIS algorithm is based on finding the best solution, where the best option is nearest to the ideal solution and farthest to the inferior anti-ideal solutions (Tzeng & Huang, 2011). The MCDA problem is typically defined by an M×N matrix ${{\rm Q}_{{\rm j\; }}}$ called an analysis matrix. The element ${{\rm q}_{\rm i}}\;$ matches the performance value of the i option regarding the decision criteria ${\rm \; }{{\rm c}_{\rm j}}$, such as represented by Eq. (16).

(16) $${{\rm Q}_{{\rm j\; }}} = \mathop {\mathop {\left[ {\matrix{ {{{\rm q}_{11}}} & \cdots & {{{\rm q}_{{\rm n}1}}} \cr \cdots & \cdots & \cdots \cr {{{\rm q}_{1{\rm m}}}} & \cdots & {{{\rm q}_{{\rm nm}}}} \cr } } \right]}\limits}\limits^{{{\rm C}_1} \ldots \ldots \ldots \ldots \ldots \ldots {{\rm C}_{\rm n}}}$$

The TOPSIS algorithm can be summarized as:

**The first step:** the normalization of the analysis matrix Q to ${\rm \; }{{\rm Q}^{\rm \prime}}$, the normalized value ${{\rm r}_{{\rm i},{\rm j}}}\;$ of each performance ${{\rm c}_{{\rm i},{\rm j}}}\;$ is calculated as follows:

(17) $${r_{i,j}} = \displaystyle{{{q_{i,j}}} \over {\sqrt {\mathop \sum \nolimits_{j = 1}^m {q_{i,j}}^2} }}$$

**The second step:** the determination of the Ideal ${{\rm p}_{ + {\rm d}}}$ and Anti-ideal ${{\rm p}_{ - {\rm d}}}$ solutions using the decision matrix for each criterion. The formulas to compute ${{\rm p}_{ + {\rm d}}}$ and Anti-ideal ${{\rm p}_{ - {\rm d}}}$ for a maximization problem are:

(18) $$\left\{ {\matrix{ {{p_{ + d}} = max\left( {{r_{i,j}}} \right)} \cr {{p_{ - d}} = max\left( {{r_{i,j}}} \right)} \cr } } \right.$$

**The third step:** the calculation of the distances of each alternative (the normalized value ${\rm \; }{{\rm r}_{{\rm i},{\rm j}}}$) from the Ideal and Anti-ideal solutions, according to the equation below:

(19) $$\left\{ {\matrix{ {{{\rm S}_{ + {\rm i}}} = \sqrt {\mathop \sum \limits_{{\rm j} = 1}^{\rm m} {{\left( {{{\rm r}_{{\rm i},{\rm j}}} - {{\rm p}_{ + {\rm d}}}} \right)}^2}} } \cr {{{\rm S}_{ - {\rm i}}} = \sqrt {\mathop \sum \limits_{{\rm j} = 1}^{\rm m} {{\left( {{{\rm r}_{{\rm i},{\rm j}}} - {{\rm p}_{ - {\rm d}}}} \right)}^2}} } \cr } } \right.$$

**The fourth step:** the computation of the relative closeness ${C_{i + }}\;$ of each solution to the ideal solution according to the following equation:

(20) $${C_{i + }} = \displaystyle{{{{\rm S}_{ - {\rm i}}}} \over {{{\rm S}_{ + {\rm i\; }}} + {{\rm S}_{ - {\rm i}}}}}$$

**The fifth step:** Sort the options in ascending order according to the ${{\rm C}_{{\rm i} + }}$ value.

### Our proposal

Multiple factors may impact the search for and selection of appropriate sensors that match the users’ requirements in the Internet of Things environment. These include finding accurate sensors, optimizing time-consuming tasks for users, and managing system performance against the dynamicity of IoT. Therefore, we suggested a new method to search for and select an appropriate service considering the above-mentioned criteria simultaneously.

### System architecture

To improve the performance of sensor search in IoT; including time-consumption and dynamicity, we propose classifying the current sensors in the network into three semantic categories and create several SSNTs (temperature, pressure, vibration, light, weather, *etc*.) in each category. The server extracts the semantic category, type, and values of the different properties from the user query. Next, the server sends them to the gateways. In each SSNT, sensors are clustered by the Whale-based Sensor Clustering Algorithm based on their context properties (accuracy, reliability, cost, availability, energy, *etc*.) before forwarding the queries to those related SSNTs. Consequently, the relevant cluster to the requested sensor properties is selected.

In order to ensure the parallel nature of IoT architecture and to minimize the network traffic caused by the dynamic context properties and number of sensors we chose a distributed architecture. Context properties are then stored in the local gateway and they are not globally updated.

The proposed architecture consists of three components: sensor, gateway, and server.

**The sensors:** these contain information and context properties and are registered within the gateways.

**The gateway:** this is responsible for managing a local network composed of three semantic categories of sensors clustered in the area of interest, and for connecting to the server.

**The server:** this is capable of processing users’ requests, connecting to several gateways to obtain the local response from each gateway (local search and selection phases), and returning the response to the user (global search and selection phases).

**Figure 5:**
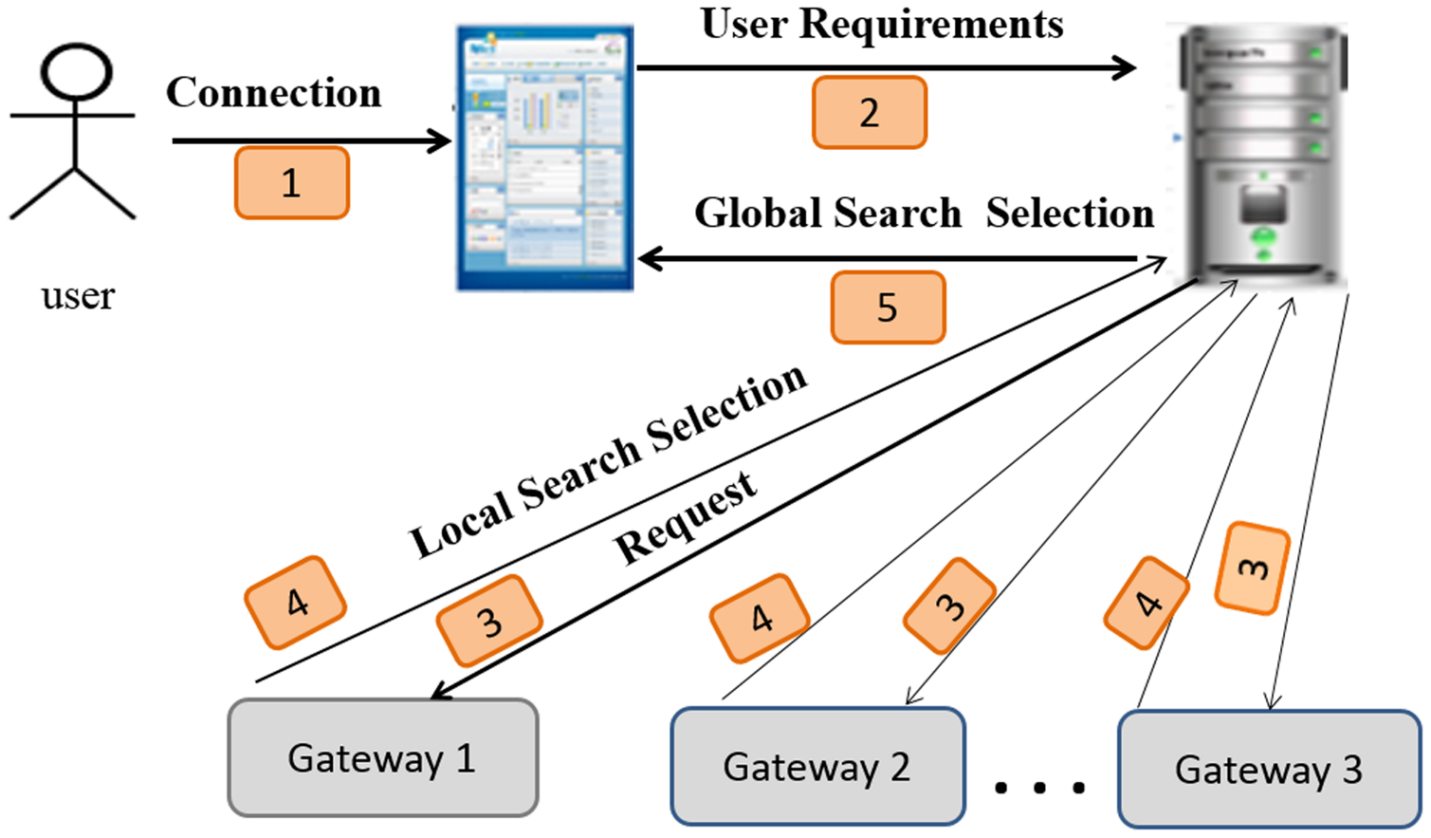
Sensor search architecture.

Figure 5 depicts an overview of the system architecture and shows the following steps:

**Connection:** the user connects to the system and expresses its requirements *via* a system interface.

**User requirements:** the user’s system sends the request (the user ID and user requirements) to the server.

**Figure 6:**
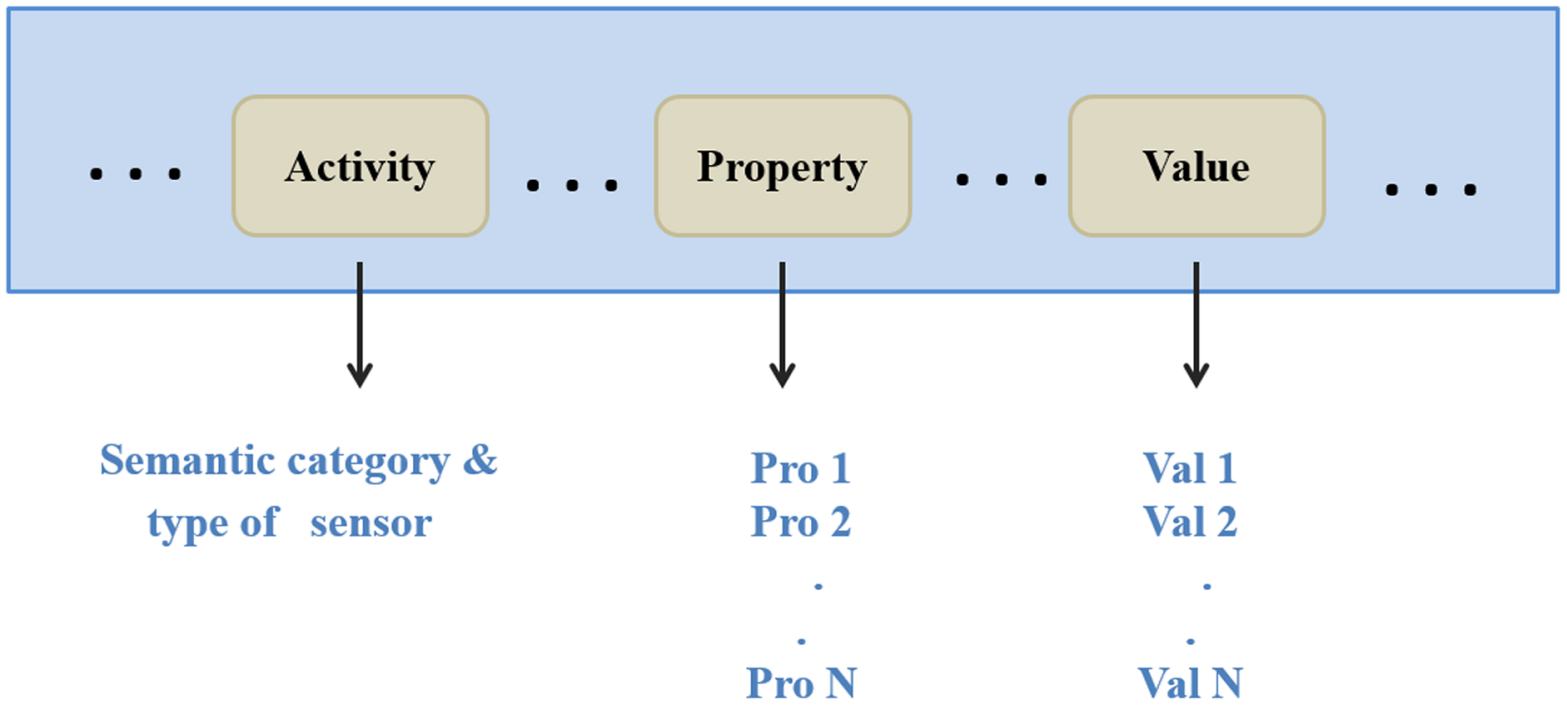
The request decomposition.

**Request:** the server can extract the semantic category, type of the requested sensor, and users’ requirements from a request. Next, it forwards them to the gateways. Figure 6 depicts a decomposed query (request).

**Local search and selection:** using the clustering process, each gateway determines the corresponding service according to the semantic category, type, and users’ requirements from a request. The adequate service to the request is returned to the server. The details of this phase are described in the “Creation of SSNTs” section.

**Global search and selection:** the server determines the gateways that can provide the appropriate service (sensor) for each request.

### Semantic modeling

**Figure 7:**
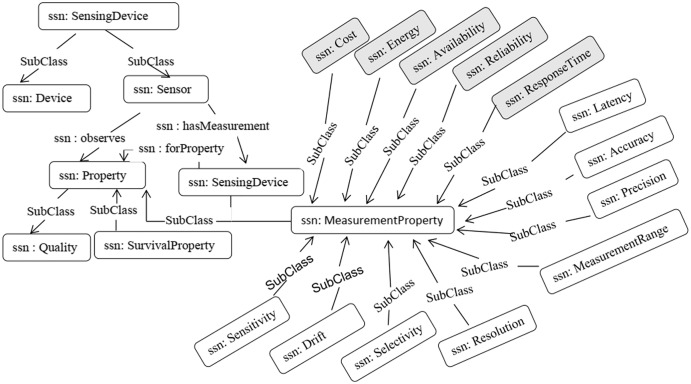
SSN ontology from a sensor perspective.

We adopted the SSN Ontology (Compton et al., 2012) to model the sensor descriptions and context properties, and to publish the information of sensors in a standard format. Compton et al. (2009) presented a comparison of different semantic sensor ontologies is presented. The SSN ontology provides the most common context properties, such as accuracy, precision, drift, sensitivity, selectivity, measurement range, detection limit, response time, frequency, and latency. Figure 7 illustrates a segment of the SSN ontology used in our work.

### Creation of SSNTs

The application of IoT can be categorized into three semantic categories (SCs) based on their focus (Sundmaeker et al., 2010; Atzori, Iera & Morabito, 2010): industry, environment, and society. For example: transportation and logistics (Yuqiang, Jianlan & Xuanzi, 2010), supply chain management (SCM) (Chaves & Decker, 2010), and aerospace, aviation, and automotive are some industry-oriented applications of IoT. Agriculture and breeding (Shifeng et al., 2011), disaster alerting, recycling, and environmental monitoring (Jang, Healy & Skibniewski, 2008) are some examples environment-oriented applications. Smart building (Minoli, Sohraby & Occhiogrosso, 2017), telecommunication, medical technology (Lu & Liu, 2011), healthcare, media, ticketing, and entertainment are some society-oriented applications.

**Figure 8:**
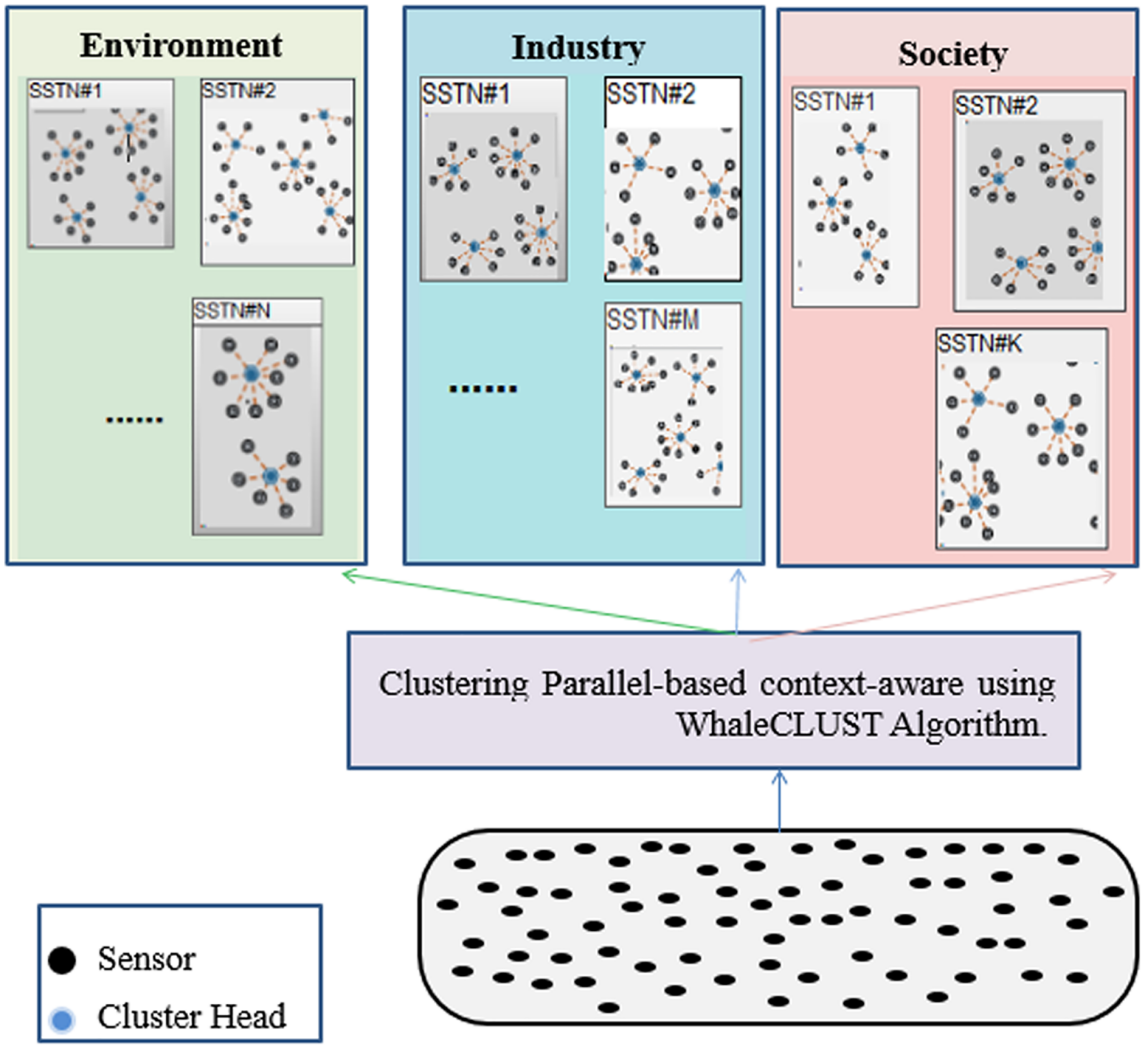
Parallel-based context-aware clustering using the WhaleCLUST algorithm in one of the gateways.

We propose an unsupervised and a decentralized creation of SSNT to improve the efficiency of routing the queries to the appropriate SSNT (type). The SSNT is based on context properties (accuracy, reliability, energy, availability, cost, *etc*.) for the three SCs (environment, society, industry) in one of the gateways (Fig. 8). For this reason, we recommend a new bio-inspired meta-heuristic that mimics the bubble-net hunting and foraging behavior of a humpback whale (WhaleCLUST-A).

We assume that a given SSNT consists of N sensors, which are described by real-value m dimensional vector properties as follows:

(21) $${{\rm X}_{\rm i}} = \left( {{{\rm x}_{{\rm i},1}},{{\rm x}_{{\rm i},2}}, \ldots ,{{\rm x}_{{\rm i},{\rm m}}}} \right)$$

where ${{\rm X}_{\rm i}}$ is a vector property of sensor i, ${{\rm x}_{{\rm i},1}}$ denotes the value of the ${{\rm j}^{{\rm th}}}\;$ property of the ${{\rm i}^{{\rm th}}}$ sensor, and m is the number of the properties.

The WhaleCLUST algorithm has been performed on each ${\rm SSN}{{\rm T}_{{\rm i},{\rm j}}}$ to cluster sensors using their context information (availability, accuracy, reliability, response time, and cost), where i is a SC such that $i = \left\{ {1;2;3} \right\}$ and j is the index of a SSNT.

The WhaleCLUST is regarded as a global optimizer because it has more abilities than other algorithm. The algorithm has a high exploration capability due to the position updating mechanism of Whales using Eq. (14), and a high emphasized exploitation and convergence, which initiate from Eqs. (7) and (12). These equations clarify that WhaleCLUST can run away from local minima with a quick convergence. WhaleCLUST solves continuous and convex problems in addition to having a larger search area, simple structure, and is adaptable in dynamic conditions.

In particular, the mobile sensors will move from a gateway to another without any change in their context properties values. The gateway that receives the new joined sensor will apply the same search process by determining the most similar group based on the semantic category, SSNT, and distance similarity of each context property. The SSNT was obtained by the WhaleCLUST algorithm. Posteriorly, we calculated the center of each cluster h noted ${{\rm C}_{\rm h}}\;$ as follows:

(22) $${{\rm C}_{{\rm h},{\rm k}}} = \displaystyle{1 \over {{{\rm n}_{\rm h}}}}\mathop \sum \limits_{{\rm i} = 1,{\rm i} \in {\rm cluster}\left( {\rm h} \right)}^{{{\rm n}_{\rm h}}} {{\rm x}_{{\rm i},{\rm k}}}$$

where ${{\rm C}_{{\rm h},{\rm k}}}$ is the value of the attribute k in the cluster h, ${\rm \; }{{\rm n}_{\rm h}}$ is the number of sensors in cluster h, and ${\rm \; }{{\rm x}_{{\rm i},{\rm k}}}\;$ is the value of the attribute k in the sensor i. Algorithm 2 illustrates the steps for calculating the center of clusters.

**Algorithm 2:** Parallel calculation of the center of cluster.

**Input: ** N_category_: The number of categories (=3)
SC_i_: The set of categories
SSNT_i,j_: The type j corresponds to the category i.
P_h_ (SSNT_i,j_): The current *h*th cluster sensor in the SSNT_i,j_
**Output:** C_j_: Centers of all clusters in SSNT_i,j_
1. **While** (N_category_ < 3) **do**
2. **For** each SSNT_i,j_, p_h_ (SSNT_i,j_) **do**
3. Calculate center cluster of C_h_ using Eq. (22).
4. Add C_h_ to C_j_
5. **End for**
6. **End while**
7. return C_j_

### Search for the relevant sensor

Here, we present the proposed technique for searching and selecting the relevant sensors in an IoT network to a given user query. The first step classifies the existing sensors into three SCs, the SSNTs within a clustered sensor are created using the WhaleCLUST algorithm in parallel for each SC. Then, Algorithm 2 is applied to the list of the centers of clusters j is elaborated as an analysis matrix with size M×N as follows:

(23) $${{\rm Q}_{{\rm j\; }}} = \mathop {\mathop {\left[ {\matrix{ {{{\rm c}_{11}}} & \cdots & {{{\rm c}_{{\rm n}1}}} \cr \cdots & \cdots & \cdots \cr {{{\rm c}_{1{\rm m}}}} & \cdots & {{{\rm c}_{{\rm nm}}}} \cr } } \right]}\limits}\limits^{{{\rm C}_1} \ldots \ldots \ldots \ldots \ldots \ldots {{\rm C}_{\rm n}}}$$

Each element ${{\rm c}_{{\rm i},{\rm k}}}$ (the performance value) corresponds to the value of the center cluster i of attribute j (j named also criterion).

In terms of quality and response time, we improved the TOPSIS algorithm (Rahim et al., 2018). TOPSIS was applied in each center of cluster of a SSNT against all the used sensors, which minimized the time consumed to select and search for the requested services.

After employing the TOPSIS algorithm for each center cluster in $\; {{\rm C}_{{\rm h},{\rm k}}}$, the best center cluster ${{\rm B}_{{\rm center}}}$ was selected; this was the most similar center cluster to the ideal sensors. Then, the list of all sensors belonging to the cluster ${\rm B}{{\rm C}_{{\rm sensors}}}$ was extracted, where the Euclidean distance between the ${\rm B}{{\rm C}_{{\rm sensors}}}$ and the ${{\rm B}_{{\rm center}}}$ was computed to select the best sensor ${{\rm B}_{{\rm sensor}}}$ between them. Algorithm 3 is shown in detail below.

**Algorithm 3:** Search and selection the best sensors.

**Input:** N_category_: The number of categories (=3)
SC_i_: The set of categories
C_j_: Centers of all clusters in SSNT_i,j_
**Output:** B_sensor_: List of centers of all clusters in SSNT_i,j_
1. **While** (N_category_ < 3) **do**
2. **For** each SSNT_i,j_ **do**
3. Apply TOPSIS algorithm to C_j_
4. Add C_h_ to C_j_
5. Get B_center_: the best center cluster (higher relative closeness value)
6. **End for**
7. Compute the Euclidean distance between each BC_sensors_ and B_center_.
8. Get the B_sensor_ that has the minimal Euclidean distance with B_center_
9. **End while**
10. **return** B_sensor_

The last phase is the global search selection. Here, the server receives the responses (the selected sensors) from the gateways. It chooses the appropriate sensors based on the user requirements by calculating the distance between each suggested solution (selected sensor) and the user requirements using Eq. (3), where a sensor with the minimal Euclidian distance is selected. The final solution provided by the service is then returned.

## Experiments

### Experiments setup and data sets

We used a HP- EliteBook 8440p computer with Intel i5 CPU running at 2.40 GHz, under Windows 7 (64-bit) and 8 GB of RAM to evaluate our proposed method. Accordingly, the simulation software was written in Python3 using the WhaleCLUST algorithm and the TOPSIS search method.

It was difficult to determine some context properties related to the sensors in public datasets therefore we used a combination of real and synthetically generated data to evaluate our proposed method on a large scale. We collected datasets from the Linked Sensor Middleware (LSM) project (Digital Enterprise Research Institute (2011), the Bureau of Meteorology (Australian Government, Bureau of Meteorology (2012), and the Phenonet project (Patni, Henson & Sheth, 2010).

In all experiments, we considered five context properties (availability, accuracy, reliability, response time, and cost) and 10,000 sensors. We took the averages of some experimental results and the parameters used in the WhaleCLUST model are shown in Table 5.

**Table 5:** The parameters used in our model.

Parameters	Values
Number of iterations (t)	600
Number of search agents	50
Number of clusters (k)	3

We created a model for the proposed architecture and datasets to evaluate the robustness, efficiency, and parameter impact of our proposed approach. We also explained the results obtained from the different experiments. Finally, we compared AntCLUST (Ebrahimi et al., 2017) and ParticuleCLUST (Wang et al., 2017) as state-of-the-art methods. Our proposed method was performed with the highest accuracy and lowest processing time required to search and select the suitable sensors to the users’ requirements.

## Results and discussion

**Figure 9:**
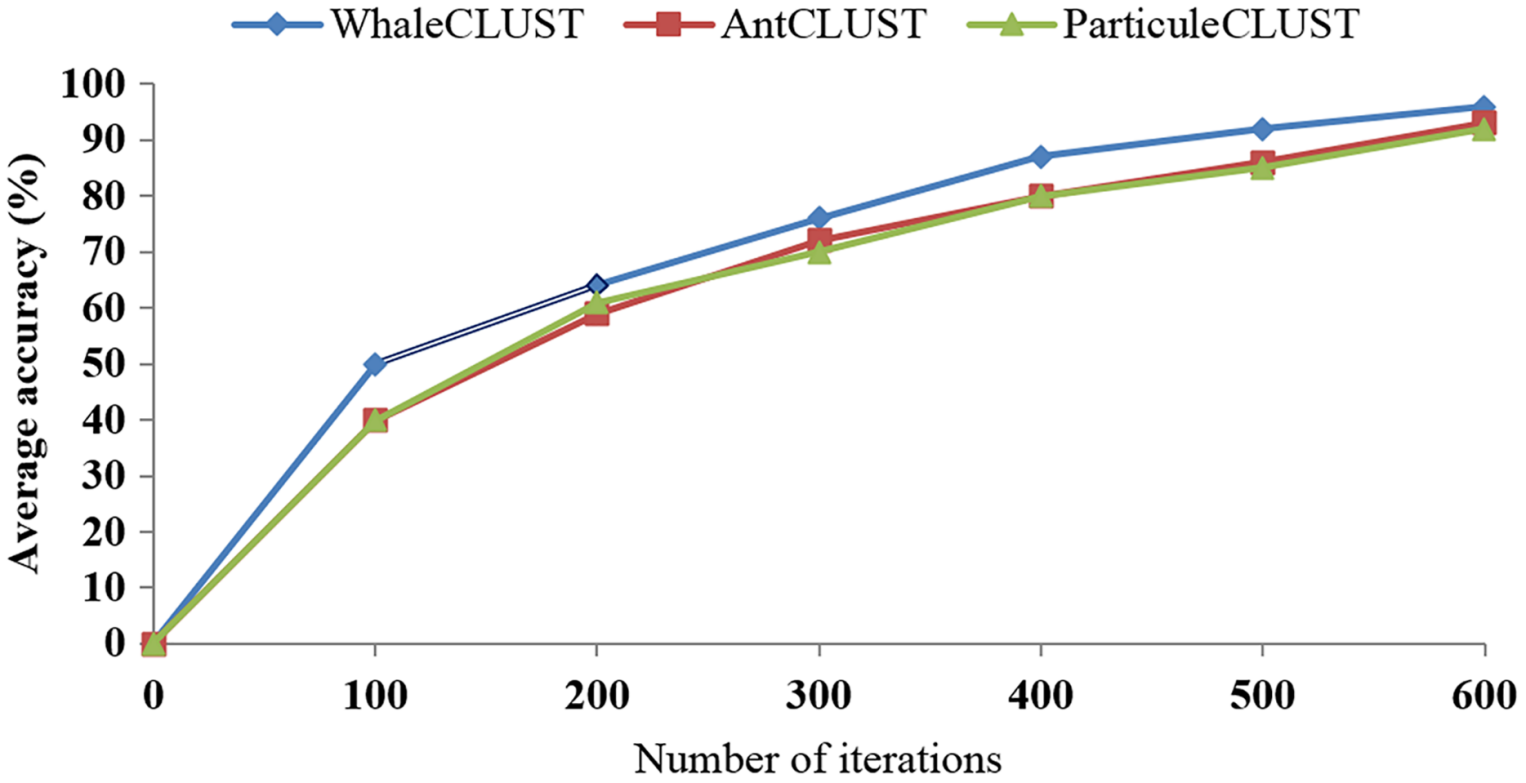
Accuracy comparisons of WhaleCLUST, AntCLUST, and ParticuleCLUST according to the number of iterations.

**Figure 10:**
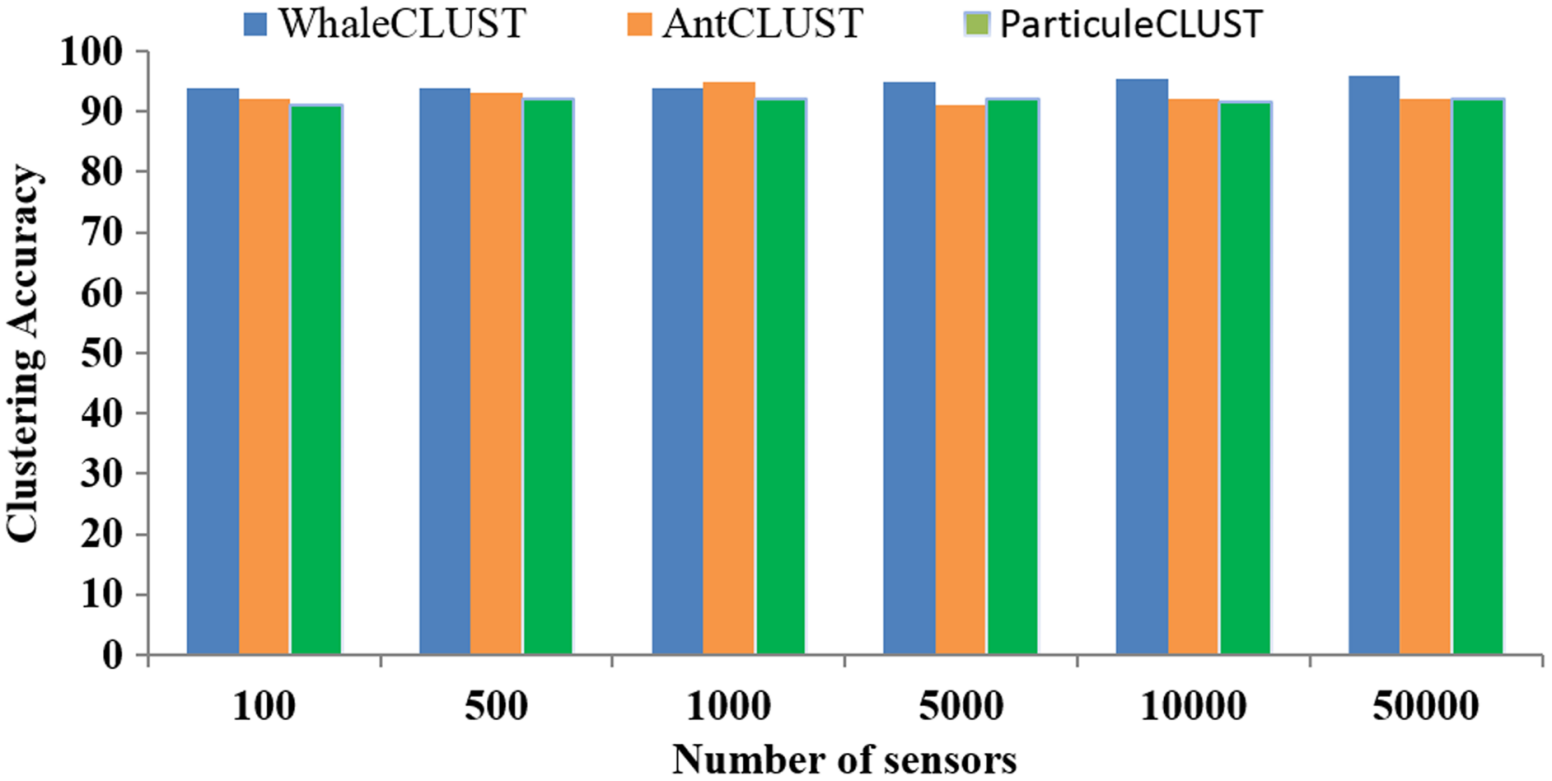
Accuracy comparisons of WhaleCLUST, AntCLUST, and ParticuleCLUST according to the number of sensors.

We evaluated the accuracy of the WhaleCLUST algorithm and compared it with the AntCLUST and ParticuleCLUST algorithms using the number of iterations and the number of sensors. The greater the number of iterations and sensors the higher accuracy of our algorithm (Figs. 9 and 10). When the number of sensors exceeded 5,000, the accuracy of our algorithm was 96%, 92% for the AntCLUST, and 91% for the ParticuleCLUST. As a result, our algorithm was shown to be more efficient and scalable than the two other ones.

**Figure 11:**
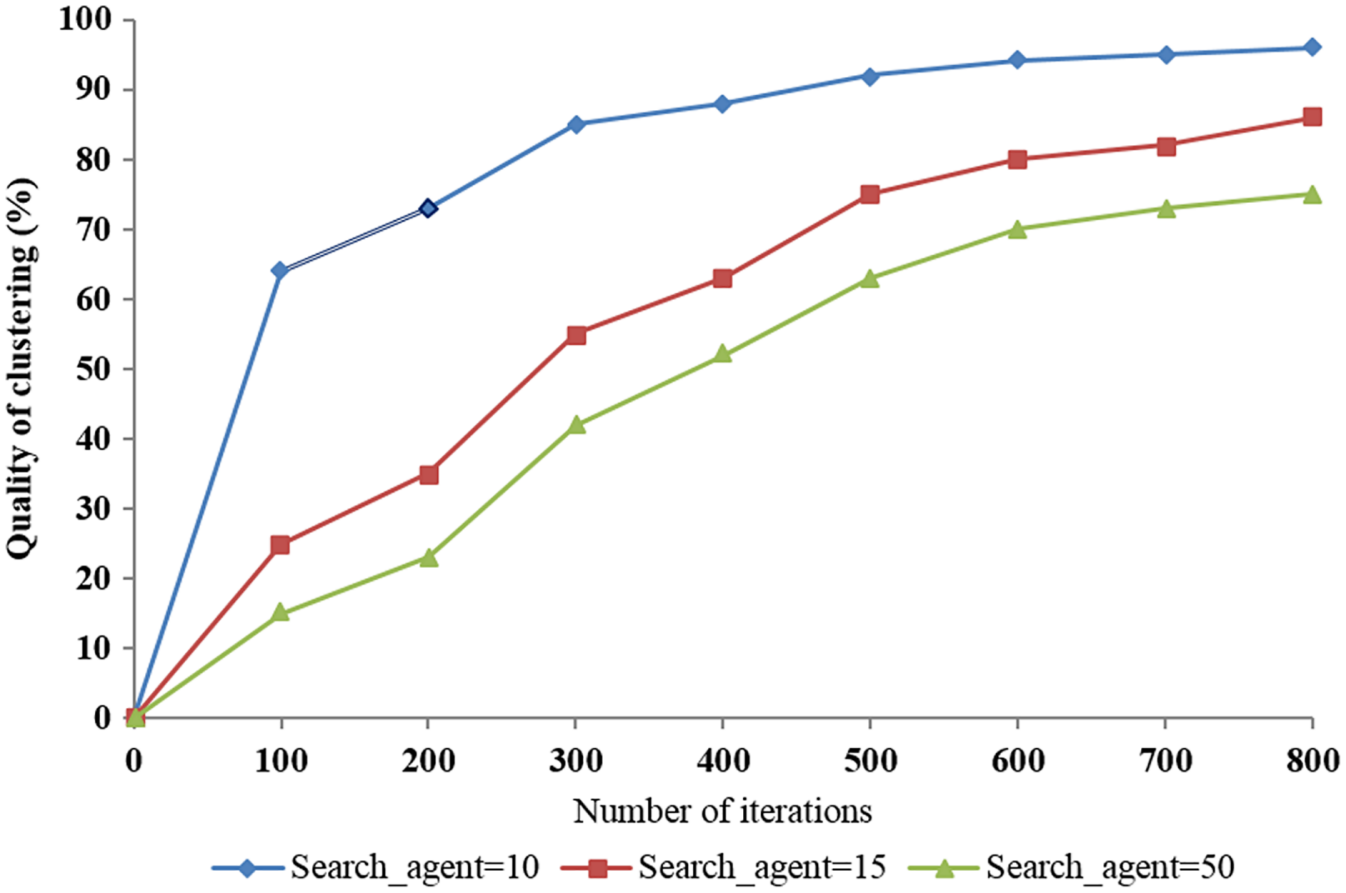
The impact of the number of search agents on the quality of clustering according to the number of iterations.

We used three trials with the number of search agents set to 10, 15, and 50 according to the number of iterations to evaluate the quality of clustering and the system performance when applying the proposed algorithm. The results show that the best quality of clustering was over 96% when the number of search agents was set to 50 (Fig. 11). This demonstrates the impact of change in the number of search agents on the quality of clustering.

**Figure 12:**
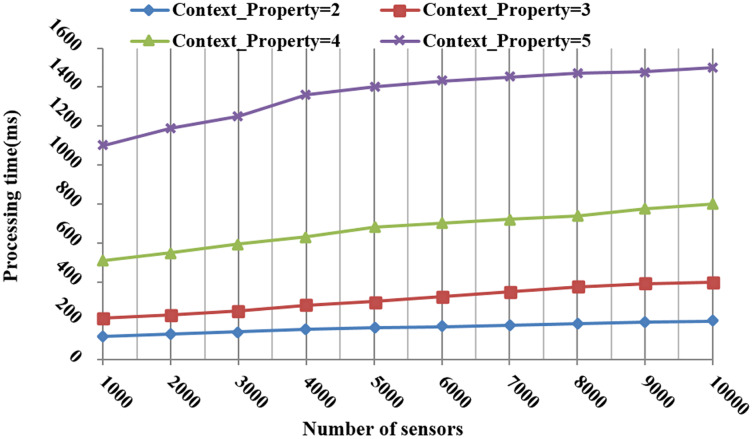
The processing time during the search and selection phases according to the number of context properties and sensors.

Figure 12 shows the processing time to respond to the request requirements with a different number of sensors and context properties. More time was consumed as the number of context properties and sensors grew. More precisely, when the number of sensors increased to 5,000, the consumed time slowly increased. This means that the search and selection tasks are efficient in a large space of interest.

**Figure 13:**
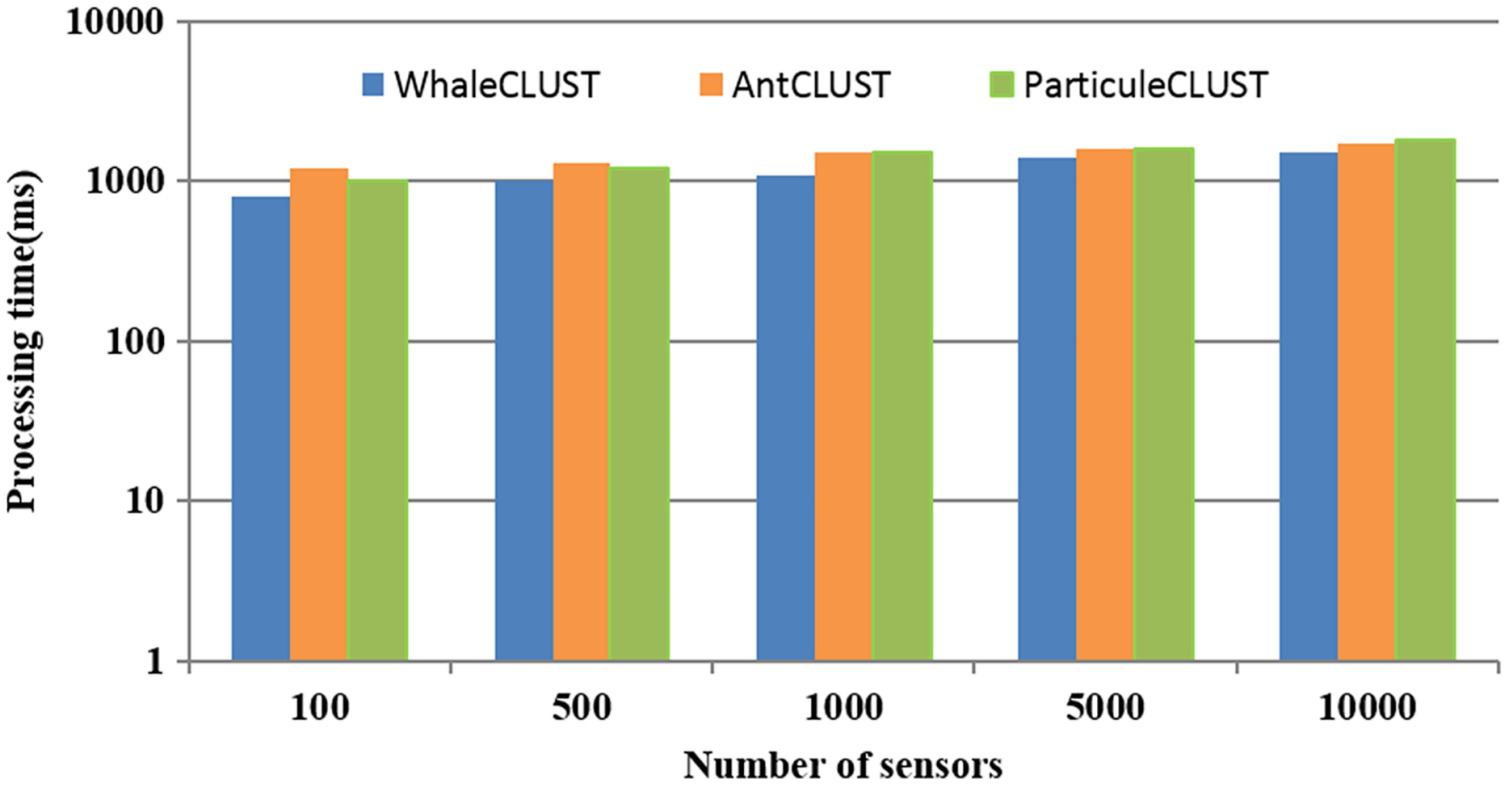
The performance of our proposed algorithm (WhaleCLUST) compared to the AntCLUST and ParticuleCLUSTalgorithms.

To demonstrate the efficiency of the search and selection phases of our approach compared to the AntCLUST and the ParticuleCLUST approaches, we measured the required processing time (Fig. 13). When the number of sensors was more than 5,000, the consumed time is semi-equal for all approaches. However, when the number of sensors was more than 5,000, the consumed was less with our approach compared to the other approaches due to the distributed model used in our approach that processes the request on a parallelized way.

## Conclusion

The advanced technology in the IoT environment allows access to multiple sensors that have similar utility. Searching and selecting the relevant and suitable sensors for user requirements is challenging. We studied the accuracy, time complexity, and scalability of the search and selection tasks to satisfy the requirements based on the context properties of sensors. We categorized the sensors into three semantic categories named environment, society, and industry. Within each SC, the SSNTs are created, where the improved Whale clustering is applied to cluster the sensors of SSNT according to the context properties. The distributed architecture consists of gateways that are connected to sensors and managed by a server, where the response is performed jointly and simultaneously among the server and the gateways. The experimental results showed that our proposed solution was promising in terms of accuracy, quality clustering, scalability, and execution time when compared to other approaches.

Furthermore, the current work can provide many benefits for practitioners and researchers who want to develop or integrate IoT applications to exploit the services provided by smart city, industry, agriculture, and healthcare systems. Our work is designed to support new technologies such as 5th generation and edge computing to efficiently and quickly respond to complex IoT requests.

We plan to apply our model in specific domains including pollution control based on storage capacity, energy consumption, and processing power. We will compare our study with works in the same context and domain and will consider the user request processing phase and the method used to extract the user requirements in future studies.

## Supplemental Information

10.7717/peerj-cs.762/supp-1 Supplemental Information 1 Code.

## Additional Information and Declarations

### Data Availability

The following information was supplied regarding data availability: Our code for clustering and searching in IoT is available in the Supplemental File.
